# Iron supplementation and iron accumulation promote adipocyte thermogenesis through PGC1α-ATGL–mediated lipolysis

**DOI:** 10.1016/j.jbc.2024.107690

**Published:** 2024-08-17

**Authors:** Xudong Mai, Yifan Liu, Jigang Fan, Lanling Xiao, Miaomiao Liao, Zhipeng Huang, Zijian Chen, Shaojun Huang, Rui Sun, Xiaowan Jiang, Liujing Huang, Jia Sun, Liwei Xie, Hong Chen

**Affiliations:** 1 Department of Endocrinology and Metabolism, Zhujiang Hospital, Southern Medical University, Guangzhou, China; 2 State Key Laboratory of Applied Microbiology Southern China, Guangdong Provincial Key Laboratory of Microbial Culture Collection and Application, Guangdong Open Laboratory of Applied Microbiology, Institute of Microbiology, Guangdong Academy of Sciences, Guangzhou, China; 3 Medical Affairs Department, Guangzhou Betrue Technology Co, Ltd, Guangzhou, China; 4 College of Life and Health Sciences, Guangdong Industry Polytechnic, Guangzhou, Guangdong, China

**Keywords:** iron homeostasis, adipose thermogenesis, stromal vascular fractions, HFE

## Abstract

Iron homeostasis is essential for maintaining metabolic health and iron disorder has been linked to chronic metabolic diseases. Increasing thermogenic capacity in adipose tissue has been considered as a potential approach to regulate energy homeostasis. Both mitochondrial biogenesis and mitochondrial function are iron-dependent and essential for adipocyte thermogenic capacity, but the underlying relationships between iron accumulation and adipose thermogenesis is unclear. Firstly, we confirmed that iron homeostasis and the iron regulatory markers (*e.g., Tfr1* and *Hfe*) are involved in cold-induced thermogenesis in subcutaneous adipose tissues using RNA-seq and bioinformatic analysis. Secondly, an *Hfe* (*Hfe*^−/−^)-deficient mouse model, in which tissues become overloaded with iron, was employed. We found iron accumulation caused by *Hfe* deficiency enhanced mitochondrial respiratory chain expression in subcutaneous white adipose *in vivo* and resulted in enhanced tissue thermogenesis with upregulation of PGC-1α and adipose triglyceride lipase, mitochondrial biogenesis and lipolysis. To investigate the thermogenic capacity *in vitro*, stromal vascular fraction from adipose tissues was isolated, followed with adipogenic differentiation. Primary adipocyte from *Hfe*^*−/−*^ mice exhibited higher cellular oxygen consumption, associated with enhanced expression of mitochondrial oxidative respiratory chain protein, while primary adipocytes or stromal vascular fractions from WT mice supplemented with iron citrate) exhibited similar effect in thermogenic capacity. Taken together, these findings indicate iron supplementation and iron accumulation (*Hfe* deficiency) can regulate adipocyte thermogenic capacity, suggesting a potential role for iron homeostasis in adipose tissues.

Energy expenditure is a fundamental aspect of metabolic balance, and is influenced by body composition, physical activity, and dietary preference (1). Basic thermogenesis or basal heat production, involves energy consumption in both basal metabolism and physiological states, also referred to as resting metabolic rate or resting energy expenditure (1,2). Adaptive thermogenesis, particularly under cold stimulation, reflects an organism's ability to generate heat through metabolic processes in adipocytes, utilizing chemical energy stored in the form of carbohydrates and fats (3). Enhancing adipocyte thermogenic capacity could be a promising approach to counteract metabolic imbalances associated with obesity and type 2 diabetes (4, 5, 6).

Mitochondrial biogenesis and function are central to the maintenance of adipocyte thermogenic capacity, where both iron-sulfur clusters and heme proteins play critical roles during this biological process. These components are instrumental in mitochondrial processes, including the tricarboxylic acid (TCA) cycle and fatty acid β-oxidation (7). Cellular iron regulation is mediated through a coordinated system involving iron transport receptors, storage proteins, pumps, and sensing complexes (8,9). Serum transferrin (Tf), the primary iron carrier, exists in iron-free (Apo-Tf) and iron-bound (Holo-Tf) forms, with Holo-Tf facilitating iron uptake *via* transferrin receptors (Tfr) (8). The interaction between Tfr1, hemochromatosis protein (HFE), bone morphogenetic protein receptors, and hematopoietin forms a complex that regulates hepcidin expression, a key modulator of systemic iron balance (8).

Previous studies have underscored a close association between iron homeostasis disruption and chronic metabolic diseases like obesity, type 2 diabetes and hepatic fibrosis (10,11). Obese adolescents in some regions, for instance, the prevalence of iron deficiency and iron deficiency anemia is notably higher than normal-weight counterparts, with a negative correlation between transferrin saturation and body mass index (12). Obese children in some regions exhibited a lower serum iron levels and transferrin saturation, alongside elevated serum hepcidin and leptin (13).

The perturbation of iron levels has profound implications for adipose tissue metabolism and plasticity. Our previous study and others found adipose-specific ablation of Tfr1 results in impaired thermogenic capacity in brown or beige adipocytes (14,15). Meanwhile, our previous work also indicated that iron deficiency induced in a model lacking transmembrane serine protease 6, exhibits impaired thermogenesis in beige or brown fat (14). Conversely, increased iron stores, through hepcidin absence, can attenuate insulin resistance and inflammation in adipose tissue induced by a high-fat diets (16). *Hfe* deficiency, primarily impacting liver iron homeostasis (17), also affects other organs including spleen (18), muscle (19), and adipose tissues (20,21). Studies have shown that iron accumulation affects fatty acid oxidation in muscle tissues of *Hfe* KO mice (19). However, the effects of increased iron storage due to *Hfe* deficiency on energy metabolism in adipose tissue remain underexplored. Our study, through transcriptomic analysis and cellular extracellular flux assessments in combination of *in vivo* and *in vitro* models, demonstrates that both ferrous ammonium citrate (FAC) supplementation and *Hfe* deficiency—induced iron storage can regulate thermogenic capacity in adipocytes and stromal vascular fractions (SVFs) of adipose tissue.

## Results

### Iron supplementation regulates energy consumption in SVFs and primary adipocytes

**Figure 1:**
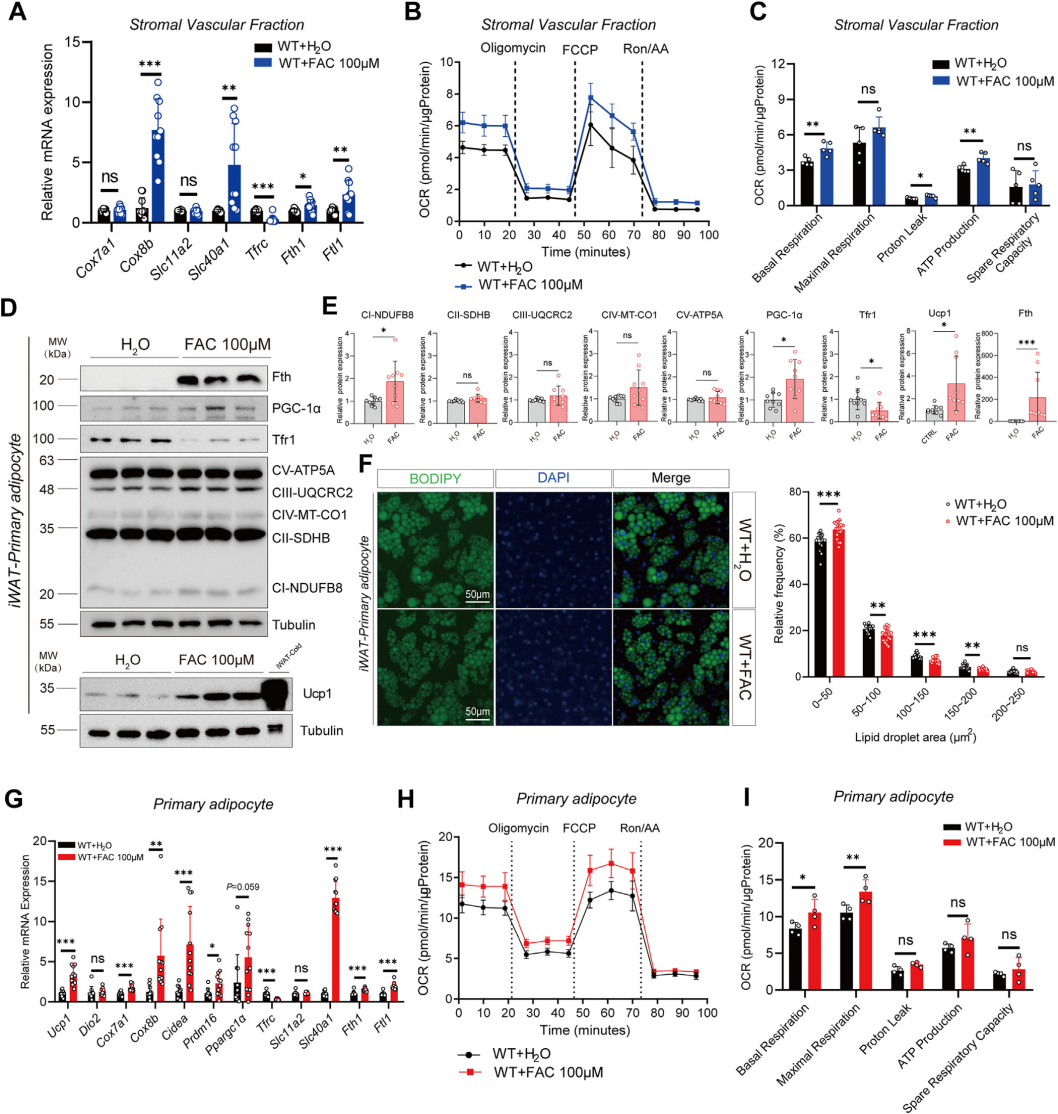
**Iron supplementation regulates energy consumption in stromal vascular fractions and primary adipocytes.***A*, qPCR analysis of iWAT SVF between H_2_O and FAC 100 μM group (n = 10/group). *B*, representative oxygen consumption rate of iWAT SVFs in Mito-stress test between control and FAC 100 μM group (n = 5/group). *C*, assay parameters of the Mito-stress test in iWAT SVFs (n = 5/group), similar result obtained in three independent experiments. *D*, representative immunoblots of mitochondrial complex, Ucp1, Tfr1, Fth, PGC-1α, and tubulin of iWAT primary adipocytes, an iWAT-cold tissue sample as a positive control for Ucp1 (n = 3/group). *E*, protein level analysis of the bands (n = 9/group), pooled from three independent experiments. *F*, representative images of fluorescence staining of iWAT primary adipocytes between control and FAC 100 μM treatment group and lipid droplet analysis (n = 15 ∼ 20 wells/group). *G*, qPCR analysis of iWAT primary adipocytes between control and FAC group (n = 11 ∼ 12 wells/group). *H*, representative oxygen consumption rate of iWAT primary adipocytes in Mito-stress test between control and FAC 100 μM group (n = 4 wells/group). *I*, assay parameters of the Mito-stress test in iWAT primary adipocyte (n = 4 wells/group), similar trends obtained in three independent experiments. Data showed as the mean ± SD. ∗*p* < 0.05, ∗∗*p* < 0.01, and ∗∗∗*p* < 0.001, N.S., no significance. Unpaired student’s *t* test for two group comparison. FAC, ferrous ammonium citrate; Fth, ferritin heavy chain; iWAT, inguinal white adipose tissue; qPCR, quantitative PCR; SVF, stromal vascular fraction; Tfr, transferrin receptor.

Investigating the impact of iron supplementation on adipocyte function, SVFs from inguinal white adipose tissue (iWAT) of *C57BL6/J* mice were isolated and differentiated into primary adipocytes. Prior to the differentiation into primary adipocytes, SVFs cell were treated with FAC at 100 μM. This treatment resulted in significant upregulation of the thermogenic gene *Cox8b*, along with notable changes in basal respiration, proton leak, and ATP production, pointing to alterations in metabolic activity due to iron supplementation (Fig. 1, *A*–*C*). Postdifferentiation, primary adipocytes with FAC-treated exhibited a pronounced increase in thermogenic markers (Ucp1 and PGC1α) and mitochondrial complex proteins (Fig. 1, *D* and *E*). This was paralleled by a reduction in lipid droplet size within the adipocytes (Fig. 1*F*). Further gene expression analysis post-FAC treatment revealed a significant elevation in thermogenic genes (*Ucp1*, *Prdm16*, and *Cox8b*) (Fig. 1*G*). Additionally, the iron-responsive gene *Tfrc* was notably downregulated following FAC supplementation, while iron storage (*Fth1 and Ftl1*) and transport genes (*Slc11a2 and Slc40a1*) showed varied responses (Fig. 1*G*). Mitochondrial stress tests conducted on these differentiated primary adipocytes indicated enhanced basal and maximal respiration rates, with no significant alterations in proton leak, ATP production, or spare respiratory capacity (Fig. 1, *H* and *I*). These findings underscore the role of iron in modulating thermogenesis and metabolic responses both in predifferentiated SVFs and in differentiated primary adipocytes, highlighting the potential of iron supplementation to influence adipocyte function and energy consumption *in vitro*.

### Role of iron in cold-induced thermogenesis in adipose tissue

**Figure 2:**
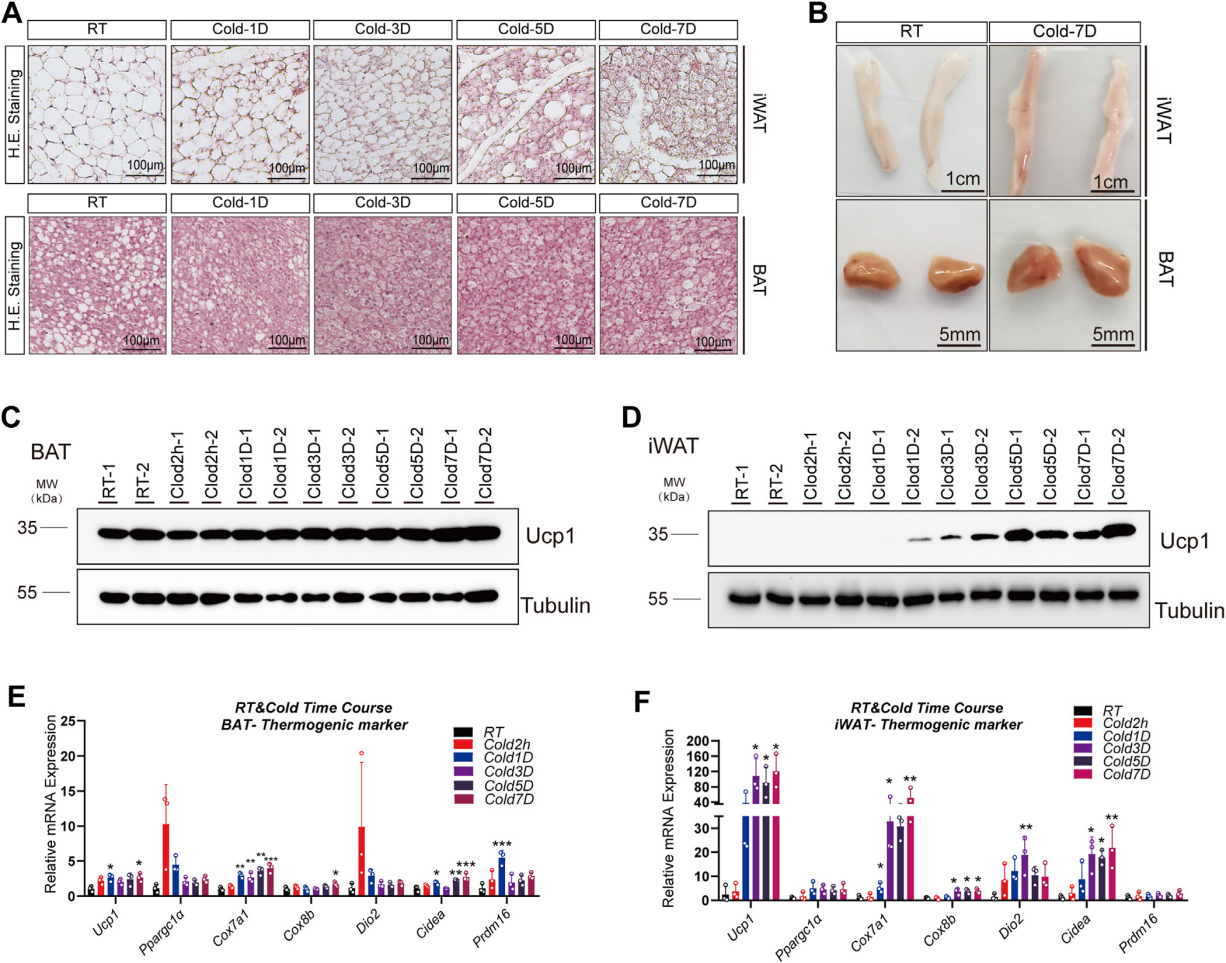
**Cold-induced thermogenesis in adipose tissues.***A*, representative H&E staining images of BAT and iWAT from 10 ∼ 15-week-old WT mice at room temperature and cold challenge. *B*, representative images of BAT and iWAT from 10 ∼ 15-week-old WT mice in room temperature and cold 7 days. *C*, Western blot results of tubulin and Ucp1 in BAT and iWAT (*D*) from 10 ∼ 15-week-old WT mice under room temperature and cold challenge. *E*, qPCR analysis of BAT and iWAT (*F*) under room temperature and cold stimulation time points (n = 3 mice/group). Data show as the mean ± SD. ∗*p* < 0.05, ∗∗*p* < 0.01, and ∗∗∗*p* < 0.001, N.S., not significant, compared with RT. Unpaired student’s *t* test for two group comparison. One-way ANOVA analysis for multiple groups comparison with a bonferroni *post hoc* analysis. BAT, brown adipose tissue; iWAT, inguinal white adipose tissue; qPCR, quantitative PCR; RT, room temperature.

**Figure 3:**
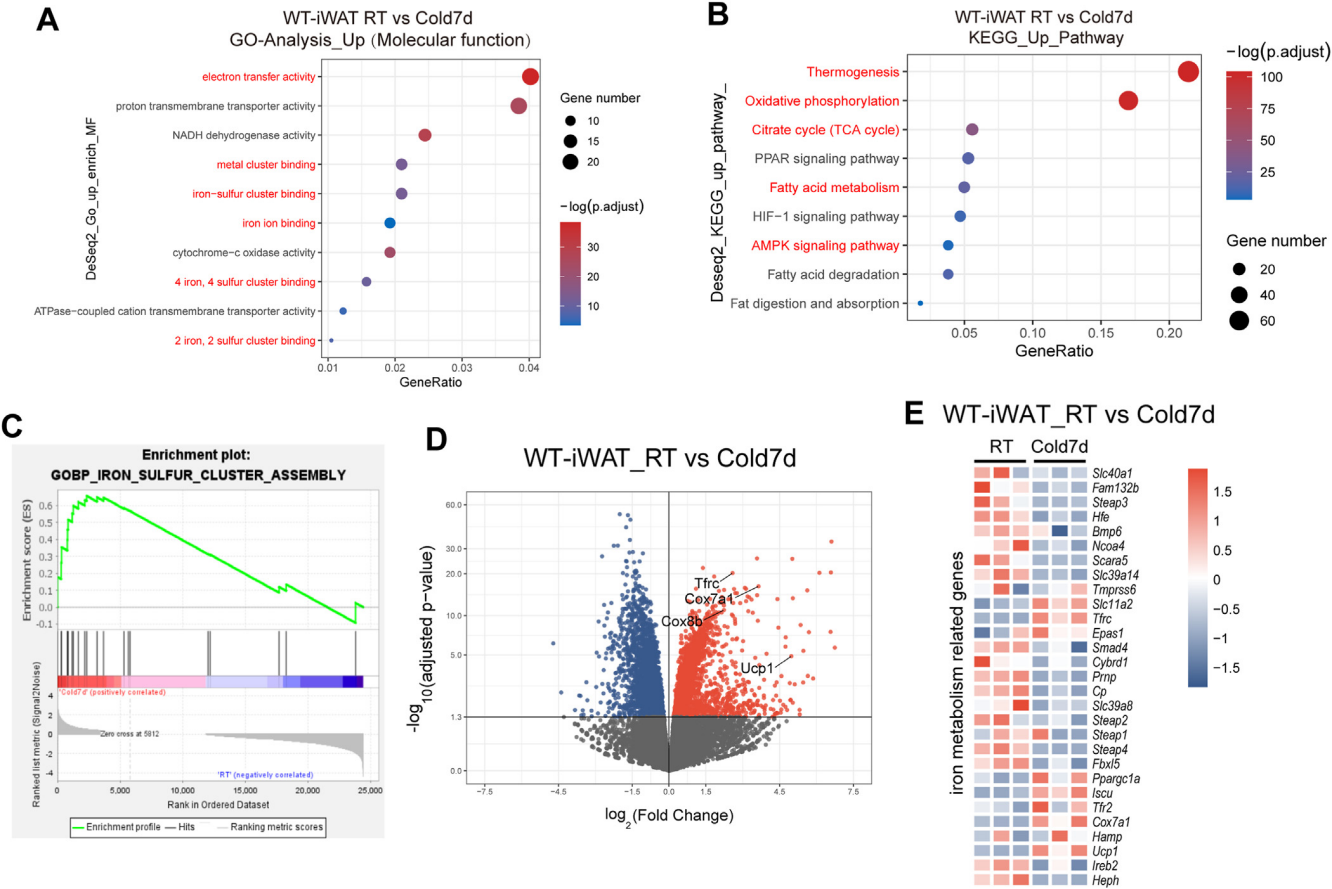
**Iron metabolism involved in cold induced adipose thermogenesis process.***A*, GO analysis of molecular function (MF) of iWAT from 10 ∼ 15-week-old WT mice between room temperature and cold exposure groups. *B*, KEGG analysis of iWAT from 10 ∼ 15-week-old WT mice between room temperature and cold exposure groups. *C*, GSEA enrich analysis of iron-sulfur cluster assembly between room temperature and cold exposure groups in iWAT in WT mice. *D*, volcano map of different expression genes in iWAT between room temperature and cold exposure groups. *E*, heatmap analysis of iron metabolism–related genes in iWAT between room temperature and cold exposure groups (n = 3 mice/group). GSEA, gene set enrichment analysis; GO, gene ontology; iWAT, inguinal white adipose tissue; KEGG, Kyoto Encyclopedia of Genes and Genomes.

**Figure S1:**
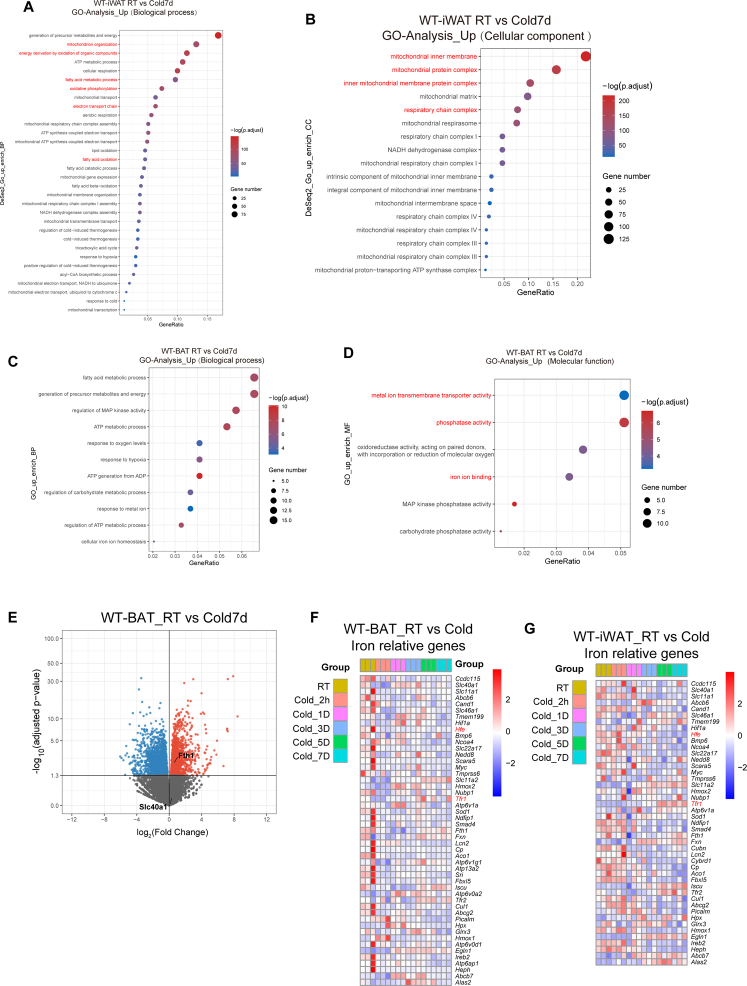


The effects of cold exposure on iron metabolism in adipose tissues were examined by analyzing brown adipose tissue (BAT) and iWAT from 10 ∼ 15-week-old *C57BL6/J* mice under room temperature (RT) and cold conditions (Fig. 2, *A* and *B*). Cold exposure led to a gradual increase in mRNA levels of thermogenic genes (*Ucp1, Ppargc1α*, *Cox7a1*, and *Cox8b*) in BAT, with a modest increase in Ucp1 protein on the seventh day of cold stimulation (Fig. 2, *C* and *E*). Similar trends were observed in iWAT, with increased mRNA and protein levels of thermogenic markers and smaller lipid droplets (Fig. 2, *A*, *D*, and *F*). RNA-seq analysis revealed enrichment in mitochondrial organization, fatty acid metabolism, oxidative phosphorylation, and fatty acid oxidation processes in iWAT postcold exposure (Fig. S1*A*). Cellular component and molecular function enrichment analyses indicated upregulated gene enrichment in mitochondrial membranes, respiratory chain complexes, and iron-binding molecules (Figs. 3*A* and S1*B*). Kyoto Encyclopedia of Genes and Genomes (KEGG) pathway analysis highlighted enhancements in thermogenesis, oxidative phosphorylation, TCA cycle, and adenosine 5′-monophosphate-activated protein kinase (AMPK) signaling pathways in iWAT post cold exposure (Fig. 3*B*), with iron-sulfur cluster enrichment (Fig. 3*C*). Similar enrichments were observed in BAT (Fig. S1, *C*–*F*). Differential gene expression analysis revealed gradually upregulated *Tfr1* and gradually downregulated *Hfe* in iWAT during cold exposure, indicating a role for iron in cold-induced adipose thermogenesis (Figs. 3, *D* and *E*, and S1*G*).

### Iron accumulation in *Hfe* deficiency modulates adipose thermogenesis

**Figure 4:**
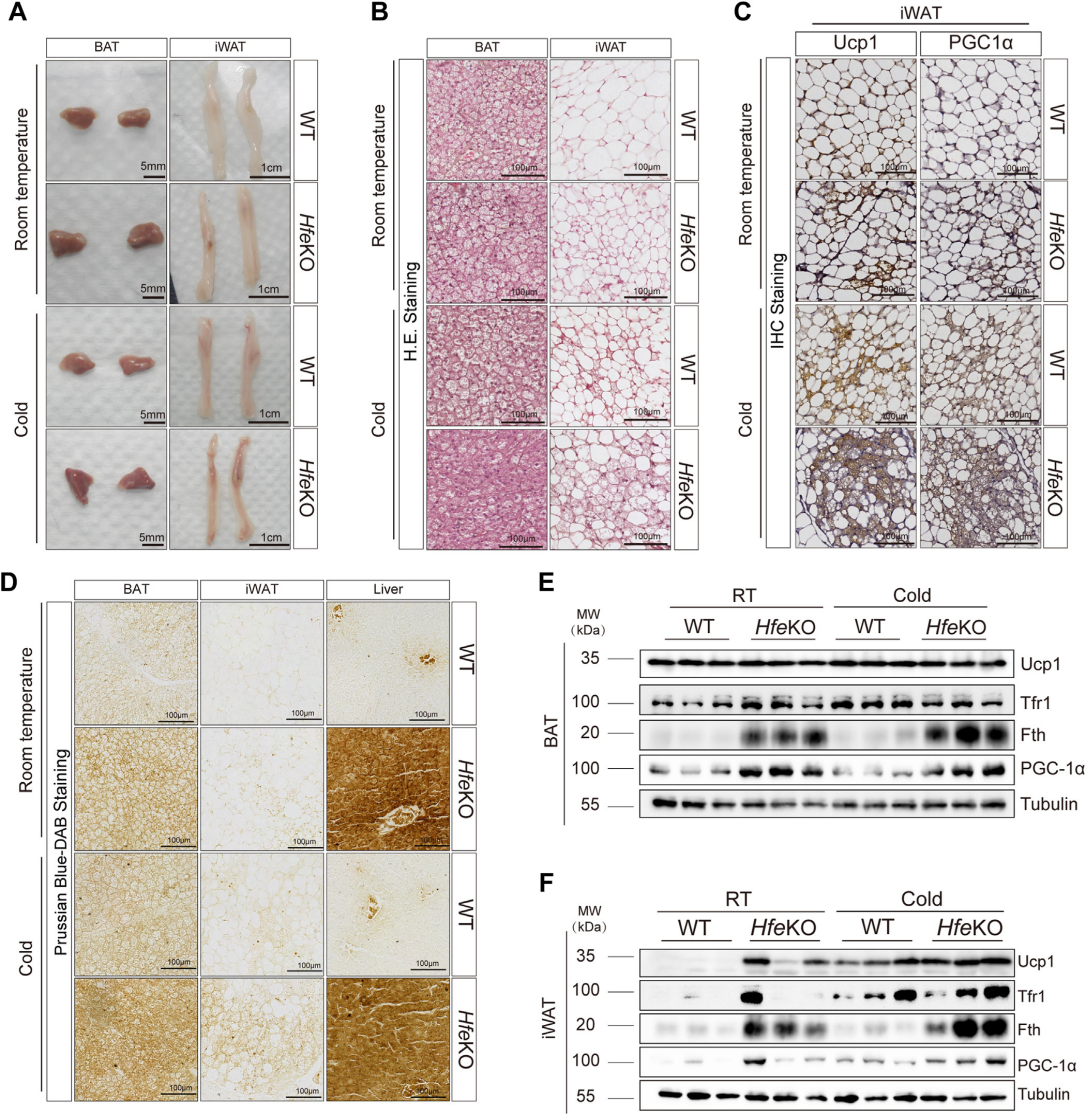
**Iron accumulation caused by *Hfe* deficiency regulate adipose thermogenesis.***A*, representative images and H&E staining (*B*) of BAT, iWAT from 30 ∼ 35-weeks-aged WT, and *Hfe*^−/−^ mice in RT and 7 days cold exposure. *C*, IHC staining (Ucp1 and PGC-1α) of iWAT from 30 ∼ 35-week-aged WT and *Hfe*^−/−^ in RT and cold exposure. *D*, representative Prussian blue-3,3′-diaminobenzidine staining images of BAT, iWAT, liver from 30 ∼ 35-week-aged WT, and *Hfe*^−/−^ in RT and cold exposure. *E*, representative protein immunoblots of Ucp1, Tfr1, Fth, PGC1α, and tubulin of BAT and iWAT (*F*) from 30 ∼ 35-week-aged WT and *Hfe*^−/−^ in RT and cold exposure (n = 3 mice/group), similar trends obtained in two independent experiments. BAT, brown adipose tissue; IHC, immunohistochemistry; Fth, ferritin heavy chain; iWAT, inguinal white adipose tissue; RT, room temperature; Tfr, transferrin receptor.

**Figure S2:**
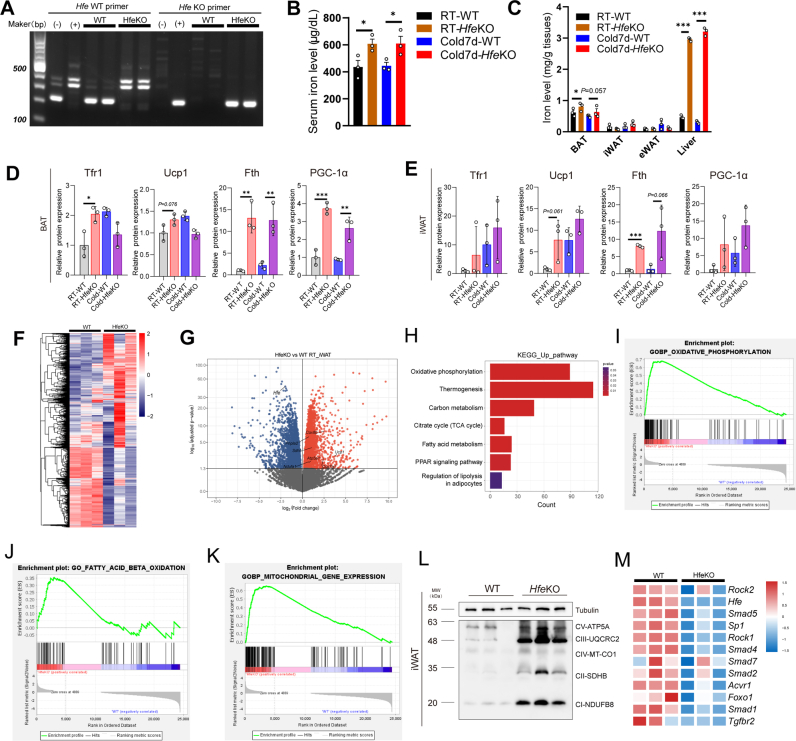


**Figure S3:**
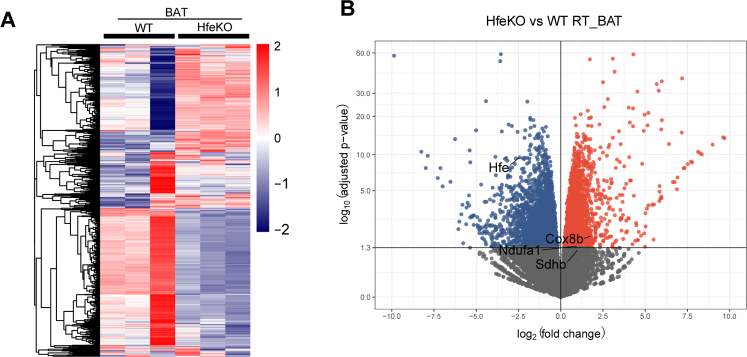


To explore the influence of iron accumulation on adipose thermogenesis, *Hfe* KO mice (*Hfe*^*−/−*^), a model of hereditary hemochromatosis with systemic iron overload, were used (Fig. S2*A*). Increased iron levels in serum, adipose tissues, and liver were observed in *Hfe*^*−/−*^ mice, compared to WT mice (Fig. S2, *B* and *C*). Smaller lipid droplets and relative higher expression of thermogenic proteins were noted in iWAT from 30 ∼ 35-week-aged *Hfe*^*−/−*^mice (Figs. 4, *A*–*F* and S2, *D* and *E*). H&E staining and immunohistochemistry staining indicated these findings (Fig. 4, *B* and *C*). Increased ferritin heavy chain (Fth) protein level in adipose tissues of *Hfe*^*−/−*^ mice indicated iron accumulation (Fig. 4, *D*–*F*). Transcriptome analysis of WT and *Hfe*^*−/−*^ mice revealed upregulation of genes associated with oxidative respiratory chain complexes in *Hfe*^*−/−*^ mice's iWAT, including *Ndufa1*, *Sdhb*, *Cox7a1*, *Cox8b*, and *Atp5a1* (Fig. S2, *F* and *G*). KEGG pathway analysis indicated enrichment in oxidative phosphorylation, thermogenesis, TCA cycle, and fatty acid metabolism in iWAT of *Hfe*^*−/−*^ mice (Fig. S2*H*), with gene set enrichment analysis indicating these findings (Fig. S2, *I*–*K*), along with the validation of protein level supporting the enhanced expression of mitochondrial respiratory chain complexes (Fig. S2*L*). Genes expression analysis also demonstrated an upregulation of *Cox8b* in *Hfe*^*−/−*^ mice's BAT (Fig. S3, *A* and *B*).

### Impact of *Hfe* deficiency and iron supplementation on SVFs and adipocytes *in vitro*

**Figure 5:**
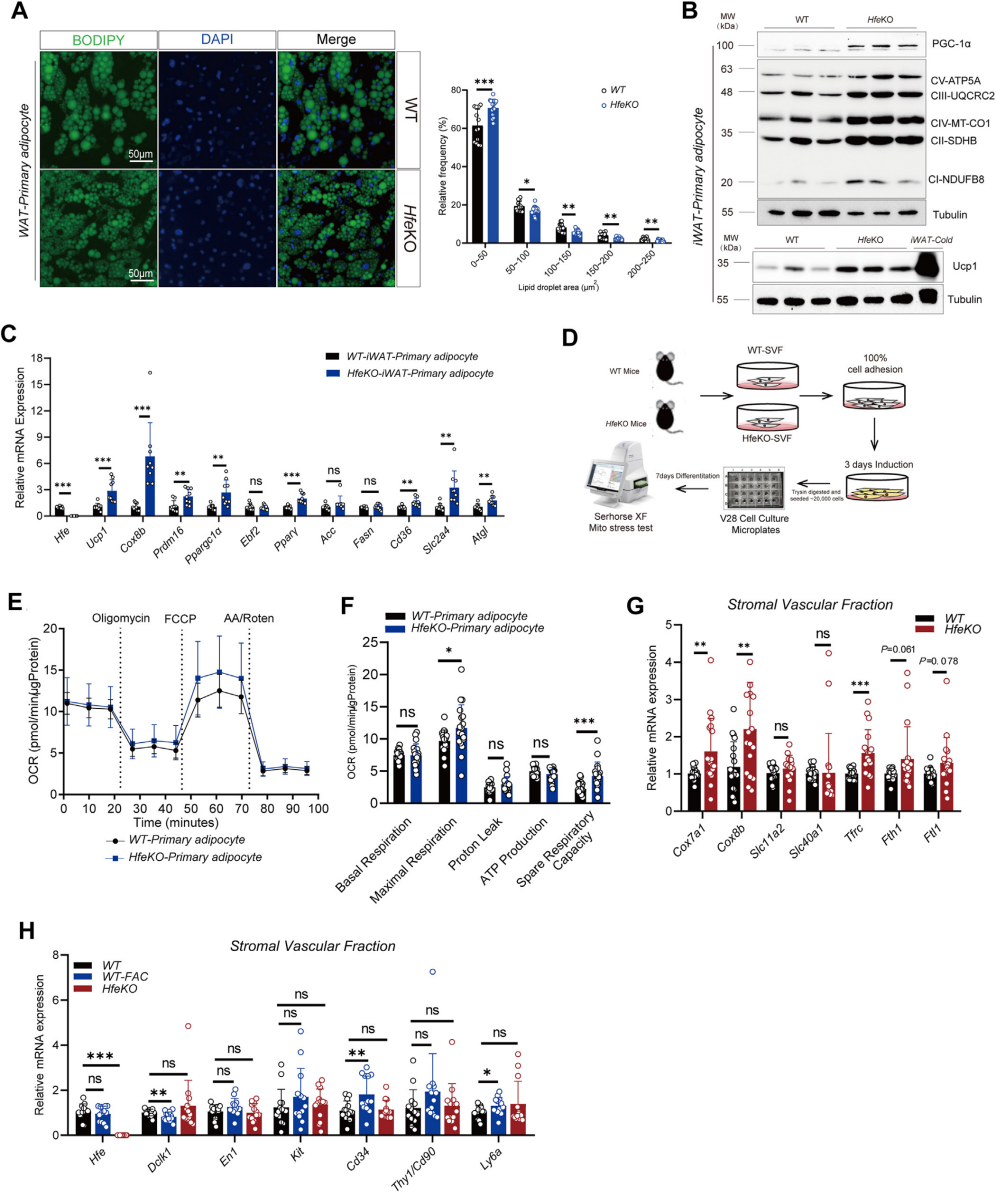
**Impact of *Hfe* deficiency and iron supplementation on SVFs and adipocytes *in vitro*.***A*, representative images of fluorescence staining and lipid droplets area analysis (n = 13 ∼ 17 wells/group) of iWAT primary adipocytes from WT and *Hfe*^−/−^. *B*, representative immunoblots of Ucp1, PGC-1α, mitochondrial complex, and tubulin of iWAT primary adipocytes from WT and *Hfe*^−/−^ (n = 3/group), similar results were seen in three independent experiments, iWAT-cold tissues sample as a positive control for Ucp1. *C*, qPCR analysis of gene expression in iWAT primary adipocyte of WT and *Hfe*^−/−^ under differentiation for 7 days (n = 9 wells/group). *D*, experimental scheme of XF Mito-stress test for primary adipocyte. *E*, oxygen consumption rate of Mito-stress test and (*F*) Assay parameters of Mito-stress test of iWAT primary adipocyte from WT and *Hfe*^−/−^ after adipogenic differentiation (n = 19 ∼ 20 wells/group). *G*, qPCR analysis of mRNA expression in SVF from iWAT between WT and *Hfe*^−/−^ group (n = 18 ∼ 19 wells/group). *H*, qPCR analysis of stem cell marker mRNA expression in SVF from iWAT in WT, WT + FAC 100 μM treatment and *Hfe*^−/−^ group (n = 13 ∼ 14 wells/group), pooled from three independent experiments. Data show as the mean ± SD. ∗*p* < 0.05, ∗∗*p* < 0.01, and ∗∗∗*p* < 0.001, N.S., not significant. Unpaired student’s *t* test for two group comparison. qPCR, quantitative PCR; FAC, ferrous ammonium citrate; iWAT, inguinal white adipose tissue; SVF, stromal vascular fraction.

**Figure S4:**
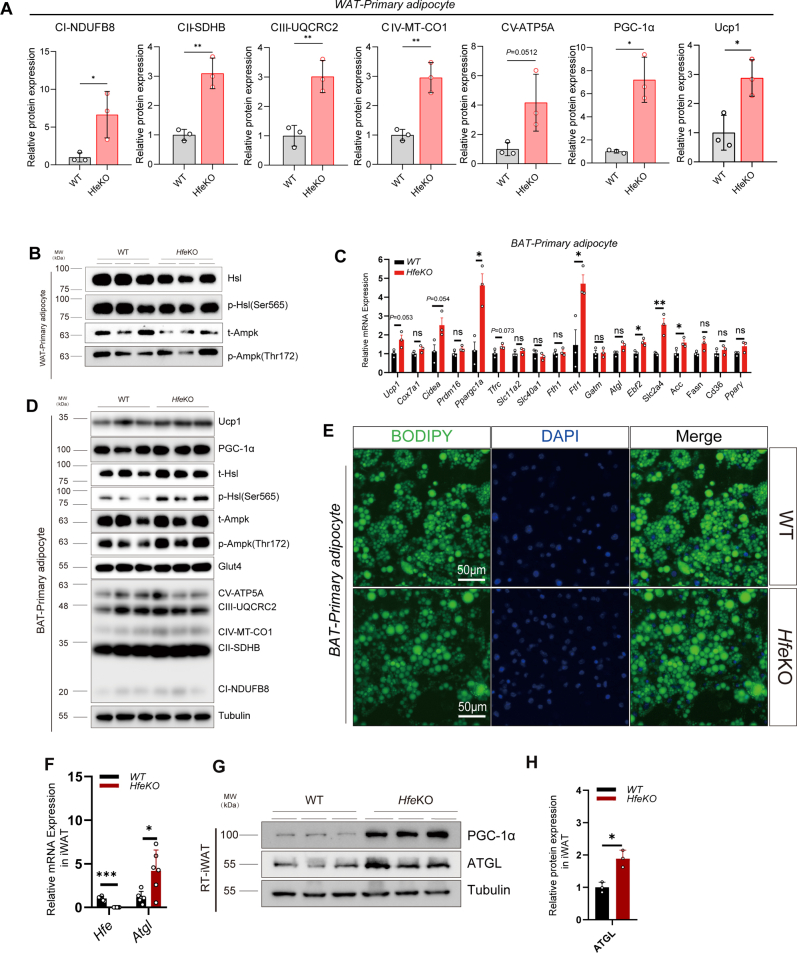


The effects of *Hfe* deficiency and iron supplementation on adipocyte thermogenesis were further assessed *in vitro* using isolated SVFs from WT and *Hfe*^*−/−*^ mice. Fluorescent staining revealed smaller lipid droplets in *Hfe*^*−/−*^ adipocytes (Fig. 5*A*). Increased protein expression of thermogenic markers and mitochondrial complexes was observed in *Hfe*^*−/−*^ adipocytes (Figs. 5*B* and S4, *A* and *B*). Quantitative PCR analysis supported the enhanced thermogenic capacity in *Hfe*^*−/−*^ adipocytes (Fig. 5*C*). Seahorse XF Mito-stress test results indicated increased maximum oxygen consumption and spare respiratory capacity in *Hfe*^*−/−*^ adipocytes (Fig. 5, *D*–*F*). Increased mRNA expression of thermogenic and iron homeostasis genes was noted in SVF cells from *Hfe*^*−/−*^ mice (Fig. 5*G*). Adipose triglyceride lipase (ATGL) is one of the important lipases on adipocyte lipolysis (22). In current investigation, we also found *Atgl* mRNA expression and ATGL protein level were increased in iWAT of *Hfe*^−/−^ mice (Fig. S4, *F*–*H*), while *Atgl* mRNA expression also increased in *Hfe*^−/−^ primary adipocytes (Fig. 5*C*). In addition, we also found BAT primary adipocytes exhibited modest increased protein levels of Ucp1 and PGC-1α in *Hfe*^*−/−*^ group, indicating elevated adipose thermogenesis (Fig. S4, *D* and *E*). Moreover, stem cell markers in SVFs revealed significant changes post-FAC treatment, indicating a potential of reprogramming of SVFs before differentiation into mature adipocyte (Fig. 5*H*).

## Discussion

Iron homeostasis disorder is closely associated with chronic diseases such as obesity and metabolic syndrome (11). However, the underlying relationships between iron accumulation (or iron overloading) and adipose thermogenesis are less investigated. In the present study, we used *Hfe*^−/−^ mice and WT mice to explore how iron accumulation is linked to heat production in adipose tissues. Employing RNA-seq bioinformatics analysis, animal and primary adipocyte models, we identified the relationship between iron homeostasis genes and adipose thermogenesis. Along with iron accumulation, *Hfe* deficiency partially modulates adipose thermogenesis and lipid metabolism.

One of our findings is that extracellular iron supplementation increased oxygen consumption and enhanced the thermogenic capacity in SVF and primary adipocytes (Fig. 1). However, it may cause adverse effects with excessive iron intervention in different cell lines (23,24). Conversely, deferoxamine , an iron chelating agent, can suppress adipogenic differentiation and reduce adipocyte thermogenic capacity and downregulate lipolysis (25). This information indicates that appropriate iron level is critical for the regulation of cell mitochondrial function and cell differentiation. Additionally, we classified multiple iron homeostasis genes involved in adipose tissues during cold-induced thermogenesis, paralleling to β3-agonist induced thermogenesis (14). Consistently, our RNA-seq data revealed an upregulation of *Tfr1*, the gene for Tfr1, during the cold exposure (Fig. 3*D*), aligning with previous studies showing Tfr1 enrichment in beige adipocytes under β-adrenergic receptor activation (14). However, homeostatic iron regulator (Hfe), known as a pathogenicity gene of hereditary hemochromatosis with multiple tissues excessive storage of iron (17), was identified to be slowly downregulated in iWAT during cold exposure based on the RNA-seq data (Figs. 3*E* and S1*G*). Besides, there may be a time lag on *Hfe* expression level in white adipose tissue, slowly downregulated in β3-agonist (CL-316,243) induced beiging and slowly upregulated upon its withdrawal (26). It is plausible that the expression of *Hfe* is associate with the thermogenic capacity of adipose tissues and its relationship between *Hfe* gene expression and thermogenesis in adipose tissues may require additional investigation.

Numerous studies have confirmed that *Hfe* is an important regulator of iron metabolism (17). Its deletion or mutation can regulate the expression of hepcidin, thereby causing a decline in the negative regulation of iron homeostasis, resulting in a state of higher iron uptake and accumulation in multiple cells and tissues (17,27). Notably, a recent study found that hepcidin (a target protein of *Hfe*) is necessary for browning capacity in white adipose tissue in mice (28). However, a previous study has reported that iron accumulation in the muscles of *Hfe*^−/−^ mice and enhanced fatty acid oxygen consumption alongside decreased glucose utilization, linked to an increased expression of carnitine palmitoyl transferase b) and decrease in pyruvate dehydrogenase) enzyme activity (19). Furthermore, increased mitochondrial respiratory capacity in *Hfe*^*−/−*^ mice's liver was noted on a normal chow diet, but not on a high-iron diet (27). Indirect calorimetry analysis also indicated a significant increases of heat production and oxygen consumption in *Hfe*^*−/−*^ mice with high-fat diet challenge (19). Other than these *Hfe*^*−/−*^ studies, it was confirmed that iron accumulation occurred in tissues and serum. Fth, a crucial subunit of ferritin and an important structure for iron storage in cells or tissues (8) and it is essential for mice energy homeostasis and adaptive thermogenesis, which was proved in mice of *Fth* deficiency (29). This is associated with iron accumulation and high expression of Fth in *Hfe*^−/−^ mice. As is well known, thermogenic markers in white adipose tissues can be dramatically promoted in cold exposure, compared with RT condition. However, aging is one of the important negative factors in regulating adipose tissues thermogenesis (30), which is also an important reason that aged mice are relatively not tolerant to cold exposure. Previous studies have found an impaired thermogenic capability during cold induced WAT beiging in middle aged WT mice (28-week-old), compared with young mice (8-week-old) (31). Interestingly, along with Fth higher expression in iWAT, we found Ucp1 and PGC-1α protein expression were higher tendency in 30∼35-week-aged *Hfe*^−/−^ mice, indicating that the iron accumulation status in *Hfe*^−/−^ mice may play an important role in white adipose tissue thermogenesis and lipid metabolism (Fig. 4). Considering that the iron accumulation level could be a one of the potential factors affecting adipose thermogenesis, it is worth further exploring in the animal models with more server iron overloaded (such as *Hjv* KO mice, *Hfe* and *Hjv* double KO mice (32)) in the future. Notably, the importance of lipolysis in energy homeostasis has been illustrated in numerous studies and the lipase play an important role in adipose lipolysis activity and lipid metabolism (22,33,34). Previous studies have been suggested that both transferrin and iron treatment can contribute to the enhanced lipolytic effect in adipocytes (35). In our study, we speculated that enhanced ATGL expression may contribute to the lipolysis in this iron accumulated status and affect the energy homeostasis (Fig. S4, *F*–*H*). SVF and adipocytes are essential parts in adipose tissues, responding to stimuli and metabolic changes (36,37).The SVF derived from adipose tissue contains abundant mesenchymal stem cells with multidirectional differentiation capacity and plasticity (38, 39, 40). Therefore, we isolated SVF from adipose tissues and induced adipogenic differentiation and significant increase in maximal respiration was observed in *Hfe*^−/−^ primary white adipocytes. This increased oxygen consumption appears to be mainly related to higher expression of Ucp1, PGC-1α, and mitochondrial complex (Fig. 5*B*).

However, it is also necessary to be noticed that the overall changes of body metabolism in *Hfe* deficiency is the result of the response of multiple metabolic organs on iron accumulation. Apart from the metabolic changes in adipose tissues and adipocytes, it is also necessary to mention that *Hfe* deficiency or mutation can increase the risk of some hepatic disease (such as hepatic fibrosis and nonalcoholic fatty liver disease, due to the cell damage and iron overloaded in liver tissue) and glucose metabolic disorders (41, 42, 43). Besides, according to dietary iron supplementation investigations, high-iron diet can induce lower fat mass, higher oxygen consumption (44), and promote fatty acid oxidation (45), but it may also lead to an impaired fasting glucose associated with insulin resistance (46). Therefore, the disadvantage effects of iron accumulation or iron dietary iron supplementation should not be ignored and it is worth more exploration and investigation to precisely regulate iron content and metabolism in different metabolic tissues.

The differentiation and development of iWATs can be traced to a subgroup of adipose vascular endothelial cells, which highly express stem cell markers. Therefore, we speculated that another potential mechanism of iron supplementation promoting cell thermogenesis may be related to cell differentiation. Here, our experiment confirmed iron supplement–enhanced mitochondrial complex genes expression and promoted oxygen consumption rates. The enhanced oxygen consumption metabolic shift was not only found in primary adipocytes but also found in SVF, a population enriched for progenitor cells. Meanwhile, this metabolic shift under iron intervention could be related to higher expressed *Cd34* in mice SVF cells (Fig. 5*H*). Previous studies have showed enhanced proliferative capacity in CD34^+^ human adipose–derived stem/progenitor cell (47), while human primary adipocyte differentiated from human adipose–derived stem/progenitor cell with high CD34 expression exhibit higher lipid turnover (fatty acid intake, lipidation, and lipolysis) (48). Interestingly, although there is enhanced oxygen consumption in *Hfe*^−/−^ primary adipocytes and higher expression of thermogenic markers (*Cox8b*) in *Hfe*^−/−^ SVF (Fig. 5), no difference was observed in the stem cell markers in *Hfe*^−/−^ SVF group under normal medium without FAC supplement. This suggests that there is other mechanism involved in regulating energy homeostasis in *Hfe*^−/−^ SVF group and it may help to identify cell subpopulations of SVF with single cell analysis in future investigation.

Moreover, recent study provided evidences that an adipose-liver tissue crosstalk in iron influx for adipose browning mediated by FoxO1-Tgfβ1 signaling (49). Interestingly, we also found Tgfβ1 signaling pathway markers were partially reduced in subcutaneous white adipose tissue in *Hfe*^−/−^ mice (Fig. S2*M*). It is plausible that tgfβ1 signaling pathway may also contribute to the thermogenesis in *Hfe*^−/−^ mice. Due to the impaired adipogenic and lipogenic effect by deferoxamine intervention, it is worth mentioning that recent work also found that iron can organize adipogenic genes expression during 3T3-L1 cell early-stage differentiation, which is mediated by the histone demethylase jumonji domain containing 1A and the DNA demethylase ten-eleven translocation 2 (50). However, considering the differences in adipogenic differentiation capacity between preadipocyte cell lines and progenitor in SVF, it is necessary to further explore the iron-dependent regulation in adipocyte differentiation and thermogenic capacity in future studies.

In summary, our work demonstrated the thermogenic promotion of iron supplementation in primary adipocytes and SVFs. We observed an enhanced thermogenic capacity in adipose tissues and primary adipocytes from the animal model with iron accumulation, suggesting a potential role for iron homeostasis in adipose function.

### Limitations of the study

Considering that in animal models of hereditary hemochromatosis (iron accumulation), in addition to *Hfe* gene knockout, there are several mouse models with *Hamp*, *Hjv*, and *Slc40a1* (coded iron pump protein) knockout. There may be different iron accumulation level among the mice models in different ages. Therefore, it is worth exploring whether there are differences in metabolic phenotypes of adipose tissues in different model of iron accumulation in future investigation.

## Experimental procedures

### Experimental animals

The *Hfe*^−/−^ mice were backcrossed with *C57BL6/J* mice, reported in the previous studies (51), kindly gifted from Dr Fudi Wang at the Zhejiang University. The mice were housed in specific pathogen-free cages, under a 12-h/12-h light and dark cycle at 23 ± 1 °C. Mice were fed a standard rodent chow diet with free access to food and water. For cold induced thermogenesis time course experiments, 10∼15-week-old male WT and *Hfe*^−/−^ mice at RT) or cold exposure (5 ± 1 °C) were sacrificed to collect the tissue samples. For middle aged mice cold challenge, 30 ∼ 35-week-old WT and *Hfe*^−/−^ mice were administered in separate cages at cold exposure (5 ± 1 °C) with sufficient food and water under light and dark cycle (12 h/12 h). After cold exposure, mice were sacrificed to collect the tissue samples for follow-up measurements. Animal experiments were approved by the Animal Care and Use Committee at Guangdong Institute of Microbiology (GT-IACUC201704071) or Animal Experiment Center of Zhujiang Hospital of Southern Medical University (LAEC-2022-004).

### Total RNA isolation and quantitative real-time PCR

The total RNA from tissues or cells were extracted with TRIzol reagent (Thermo Fisher Scientific), according to the manufacturer’s instruction. RNA concentration was determined using the NanoDrop absorbance spectroscopy (Thermo Fisher Scientific), followed by reverse transcription to complementary DNA utilizing the 5× All-In-One Master Mix (G490, AbmGood). Complementary DNA was used to determine the gene expression using the SYBR Green Master Mix (A25778, Applied Biosystems) on a QuantStudio 6 Flex Real-Time PCR System (Thermo Fisher Scientific). The gene expression was normalized to the expression of 18S ribosomal RNA. All primer sequences for quantitative PCR were listed in Table S1.

### Protein isolation and Western blot analyses

Total protein from tissues or primary cells were extracted with radio immunoprecipitation assay buffer, supplemented with 1× proteasome inhibitor cocktail (Thermo Fisher Scientific). The protein concentration was determined by using bicinchonininc acid protein assay kit (Thermo Fisher Scientific). The protein samples were separated using PAGE and transferred to polyvinylidene fluoride membranes (Merck Millipore) with precooled transfer buffer. Polyvinylidene fluoride membrane with proteins was blocked with 5% (w/v) nonfat milk at RT for 1 h, followed by overnight incubation with primary antibodies: (Tubulin: sc-365791, Santa Cruz); (PGC1α: AB3242, Millipore); (Ucp1:ab10983, Abcam); (total-HSL, #4107, p-HSL(Ser565), #4137, Cell Signaling Technology, CST.); (total-AMPK, #5831, p-AMPK (Thr172), #2523, CST.); (ATGL:#2138, CST.); (Glut4: A7637, Abclonal); (Tfr1: ab84036, Abcam); (Fth: sc-376594 Santa cruz); and (Mito-complex antibody: ab110413, Abcam) on a rocker overnight in 4 °C refrigerator and the secondary antibodies were used for incubation at RT for 1 h. Bands were visualized using enhanced chemiluminescence reagents (Abclonal) using the ChemiDoc Imaging System (Bio-Rad). Protein expressions data were quantified by using ImageJ software (https://imagej.net/).

### Tissues and serum iron estimation

Non-heme iron levels of adipose tissues, liver, and serum iron levels were detected following a standard ferrozine assay protocol as described before (14,52). Briefly, tissues were homogenized with ddH_2_O, followed by the addition of equal volume of 10% trichloroacetic acid in 3 M HCl. After 1 h digestion at 100 °C, 50 μl of each sample and iron standard were incubated at 37 °C for 1 h with equal volume ferrozine working solution (50 μl 100 mM ferrozine, 72 μl 70% mercaptoacetic acid, 2.5 ml 3 M sodium acetate in 5 ml total volume with ddH_2_O). After incubation, absorbance (*A*) value at 562 nm was measured by a microplate photometer (Thermo Fisher Scientific). The individual wet tissues weight was used to normalize the iron level.

### RNA-seq and bioinformatics analysis

Total RNA samples were sequenced using a BGI-SEQ2500 platform (Beijing Genomics Institute). The high-quality RNA-seq reads were further aligned to the mouse genome (GRCm38/mm10) using HISAT2 and assembled against mouse mRNA annotation using high-throughput sequencing on a high-performance computational system. Differentially expressed genes (DEGs) were analyzed using the DESeq2 (https://bioconductor.org/packages/release/bioc/html/DESeq2.html) package in R. Genes were considered significantly upregulated or downregulated at p-adj < 0.05. Heatmaps were generated using the heatmap package in R based on the raw count of DEGs. Gene ontology analysis was performed using the R package ClusterProfiler for DEGs (upregulated or downregulated). DEGs (p-adj < 0.05) were further analyzed using gene set enrichment analysis. Both upregulated and downregulated genes were functionally categorized using gene ontology and KEGG pathway enrichment analyses.

### SVF isolation and primary cell culture

The SVF of BAT and iWAT were isolated as previously described (53). Adipose tissue from 5∼7-week-old mice was carefully separated into enzyme working solution (1.5 mg/ml collagenase type II, 2.4 U/ml dispase II, and 10 mm CaCl_2_, filtered through a 0.22 μm filter), scissors cut to pieces. Digestion was carried out in a water bath at 37 °C for 30 min, and the mixture was observed every 10 min. The digested tissue fragments were filtered through a 40 μm sterile filter, neutralized in medium containing 10% fetal bovine serum (FBS), and centrifuged (1000×*g*, 10 min) to precipitate cells as SVF. The cells were resuspended in complete medium (Dulbecco's modified Eagle's medium [DMEM] supplemented with 10% FBS, 1% Penicillin, Streptomycin) and seeded into 24-well plates overlaid with collagen. After 5 h, the cells were rinsed with complete medium (DMEM, 10% FBS, 1% P.S) to remove tissue debris and red blood cells. Cultures were incubated in a 5% CO_2_ incubator at 37 °C. After cells 100% adherence, they were induced adipocyte differentiation. BAT primary cell culture protocol: 3 days in induction medium, a fresh complete DMEM-high glucose medium with 3-isobutyl-1-methylxanthine, 0.5 mM (Sigma: I-5879); indomethacin, 125 μM (Sigma: I7378); dexamethosone, 2 μg/ml, (Sigma: D1756); insulin, 1 μM (Solarbio, I8830); T3, 1 nM (Sigma: T2877); rosiglitazone, 1 μM (Sigma: R2408), along with differentiation medium (insulin, 1 μM, Solarbio I8830; T3, 1 nM, Sigma: T-2877; rosiglitazone, 1 μM) for 7 days culture. iWAT primary cell culture protocol: induction medium supplemented with 3-isobutyl-1-methylxanthine 0.5 mM; indomethacin, 125 μM; dexamethosone, 2 μg/ml; rosiglitazone, 1 μM, culture for 3 days, and cells induced differentiation with differentiation medium (insulin, 1 μM and T3, 1 nM) for another 7 days. FAC treatment was performed in differentiation medium for 7 days. Bodipy staining and 4′,6-diamidino-2-phenylindole staining were performed by using lipid probes (Thermo Fisher Scientific, D-3834) and Hoechst reagent (Invitrogen, H1399) before cell harvested. Images were captured by using fluorescence microscopes (Invitrogen EVOS), and adipocyte lipid droplet sizes were analyzed by using ImageJ. The analysis was performed with minor adjustments as previously described (54). Briefly, after setting the scale of the images, 8 bit images were converted into binary images and watershed for lipid object separation. The size (μm^2^) of lipid droplets were displayed by ImageJ and presented in GraphPad prism 8.0 (https://www.graphpad.com) with relative frequency.

### Oxygen consumption rate assays

After 3 days of induction, the primary cells were digested with 0.25% trypsin and seeded into seahorse V28 well plates (∼20,000 cells/well), and white SVF cells from WAT were seeded in seahorse V7-PS cell plates. Oxygen consumption rate assays were performed on the seventh day of primary adipocyte differentiation or on the third day of SVF cells with FAC treatment. Preheat the machine in advance and hydrate the probe plate overnight. Cell culture plates were rinsed three times with working assay solution (pH 7.4) (1 mM glucose, 2 mM glutamine, and 10 mM pyruvate). Finally, 500 μl working solution was added per well. The cells were incubated in a CO_2_-free incubator at 37 °C for 50 min. The hydrated probe plate was added with drugs (oligomycin 1.5 μM, carbonyl cyanide 4-(trifluoromethoxy)phenylhydrazone 4.0 μM, antimycin/rotenone 1.0 μM), and then put into the cell plate to run the XF Cell Mito Stress Test program. Data were collected and normalized with cell protein level in Seahorse Software Wave Desktop (V2.6).

### H&E staining

Fresh tissues were immersed in 10% formalin for 48 h, dehydrated, and made into paraffin-embedded sections with a thickness of 4 μm. For H&E staining, paraffin wax was melted (65 °C, 40 min), sections were deparaffinized by immersing in xylene (three times for 5 min each), and xylene was rinsed with alcohol series. The tissues were stained with hematoxylin for 3 min, differentiated in 0.3% hydrochloric alcohol for 1 ∼ 2 s, and blued with ammonia solutions. The sections were immersed in eosin, dehydrated with alcohol series, dried with xylene, and mounted with mounting medium (Macklin, 96949-21-2). Images were captured by microscope system (Hitachi) or scanned on automatic digital slice scanning system (Guangzhou Betrue Technology Co, Ltd).

### Immunohistochemistry

Paraffin slices with tissue sections at 4 μm thickness were deparaffinized in xylene and hydrated in alcohol series, followed with ddH_2_O rinsed. Soak the slides in Tris EDTA buffer in a boiling water bath for 1 h for antigen retrieval. After cooling to RT, the slides were washed with PBS two times for 5 min, followed with 0.5% Triton X-100 permeabilization for 10 min. Incubate at RT with 3% H_2_O_2_ for 10 min to eliminate endogenous peroxidase activity and wash with ddH_2_O three times. Sections were blocked in 3% bovine serum albumin containing 5% goat serum for 1 h, followed with primary antibody incubation (Ucp1, 1:500, abcam; PGC1α, 1:500, millipore) at 4 °C overnight. After incubation, the sections were washed with PBS for three times and incubated secondary antibody (1:500) in 3% bovine serum albumin containing 5% goat serum for 1 h at RT. The sections were incubated with 3,3′-diaminobenzidine (DAB) reagent for 10 ∼ 12 min. Restained the nucleus for 3 min with hematoxylin, followed with differentiator to remove excess dye. After water washing, bluing with ammonia solution for 10 min. The sections were dehydrated and cleared with alcohol series and xylene. Sections were mounted with mounting reagent and images captured and scanned as described above.

### Prussian blue-DAB staining

Prussian blue-DAB staining was as performed as described (55). Paraffin slices dewaxed, rehydrated with standard procedures, and incubated in 1% H_2_O_2_ in methanol for 10 min. After ddH_2_O washing, sections were immersed in 2% HCL and 2% potassium ferrocyanide at ratio of 1:1 for 30 min. Then sections were washed with ddH_2_O and immersed in 0.05% DAB in PBS pH 7.4 for 10 min. Sections were immersed in 0.044% H_2_O_2_ with 0.05% DAB in PBS pH 7.4 for another 10 min and washed with ddH_2_O. Then sections were dehydrated in alcohol series and dried with xylene, followed by mounting and images were captured and scanned.

### Statistical analysis

Data were presented as the mean ± SD. Statistical analysis was performed in GraphPad prism 8.0 by using the unpaired Student^’^s *t* test for two group comparison. One-way ANOVA analysis was used for multiple groups comparison, followed by a Bonferroni post hoc analysis. *p* values is shown as ∗*p* < 0.05, ∗∗*p* < 0.01, and ∗∗∗*p* < 0.001 for showing differences and the NS stands for not significant (*p* > 0.05).

## Data availability

RNA-seq sequencing data were uploaded at National Microbiology Data Center (NMDC: http://nmdc.cn/) and requests for data should be approved by H. C. (chenhong123@smu.edu.cn) and L. X. (xielw@gdim.cn). Further information and requests for resources and reagents should be directed to and will be fulfilled by the contact, H. C. (chenhong123@smu.edu.cn) and L. X. (xielw@gdim.cn).

## Supporting information

This article contains supporting information (14, 49).

## Conflict of interest

The authors declare that they have no conflicts of interest with the contents of this article.

## References

[1] Westerterp K.R., Feingold K.R., Anawalt B., Blackman M.R., Boyce A., Chrousos G., Corpas E. (2000). Control of Energy Expenditure in Humans.

[2] Heymsfield S.B., Smith B., Dahle J., Kennedy S., Fearnbach N., Thomas D.M. (2021). Resting energy expenditure: from cellular to whole-body level, a mechanistic historical perspective. Obesity (Silver Spring).

[3] Brychta R.J., Chen K.Y. (2017). Cold-induced thermogenesis in humans. Eur. J. Clin. Nutr..

[4] Cheng L., Wang J., Dai H., Duan Y., An Y., Shi L. (2021). Brown and beige adipose tissue: a novel therapeutic strategy for obesity and type 2 diabetes mellitus. Adipocyte.

[5] Li Y., Wang D., Ping X., Zhang Y., Zhang T., Wang L. (2022). Local hyperthermia therapy induces browning of white fat and treats obesity. Cell.

[6] Yin X., Chen Y., Ruze R., Xu R., Song J., Wang C. (2022). The evolving view of thermogenic fat and its implications in cancer and metabolic diseases. Signal. Transduct. Target Ther..

[7] Vercellino I., Sazanov L.A. (2022). The assembly, regulation and function of the mitochondrial respiratory chain. Nat. Rev. Mol. Cell Biol..

[8] Kawabata H. (2019). Transferrin and transferrin receptors update. Free Radic. Biol. Med..

[9] Venkataramani V. (2021). Iron homeostasis and metabolism: two sides of a coin. Adv. Exp. Med. Biol..

[10] Harrison A.V., Lorenzo F.R., McClain D.A. (2023). Iron and the pathophysiology of diabetes. Annu. Rev. Physiol..

[11] Gonzalez-Dominguez A., Visiedo-Garcia F.M., Dominguez-Riscart J., Gonzalez-Dominguez R., Mateos R.M., Lechuga-Sancho A.M. (2020). Iron metabolism in obesity and metabolic syndrome. Int. J. Mol. Sci..

[12] Manios Y., Moschonis G., Chrousos G.P., Lionis C., Mougios V., Kantilafti M. (2013). The double burden of obesity and iron deficiency on children and adolescents in Greece: the Healthy Growth Study. J. Hum. Nutr. Diet.

[13] del Giudice E.M., Santoro N., Amato A., Brienza C., Calabro P., Wiegerinck E.T. (2009). Hepcidin in obese children as a potential mediator of the association between obesity and iron deficiency. J. Clin. Endocrinol. Metab..

[14] Li J., Pan X., Pan G., Song Z., He Y., Zhang S. (2020). Transferrin receptor 1 regulates thermogenic capacity and cell fate in Brown/beige adipocytes. Adv. Sci. (Weinh).

[15] Qiu J., Zhang Z., Wang S., Chen Y., Liu C., Xu S. (2020). Transferrin receptor functionally marks thermogenic adipocytes. Front. Cell Dev. Biol..

[16] James J.V., Varghese J., John N.M., Deschemin J.C., Vaulont S., McKie A.T. (2023). Insulin resistance and adipose tissue inflammation induced by a high-fat diet are attenuated in the absence of hepcidin. J. Nutr. Biochem..

[17] Barton J.C., Edwards C.Q., Acton R.T. (2015). HFE gene: structure, function, mutations, and associated iron abnormalities. Gene.

[18] Martins R., Silva B., Proenca D., Faustino P. (2011). Differential HFE gene expression is regulated by alternative splicing in human tissues. PLoS One.

[19] Huang J., Jones D., Luo B., Sanderson M., Soto J., Abel E.D. (2011). Iron overload and diabetes risk: a shift from glucose to Fatty Acid oxidation and increased hepatic glucose production in a mouse model of hereditary hemochromatosis. Diabetes.

[20] Yue F., Cheng Y., Breschi A., Vierstra J., Wu W., Ryba T. (2014). A comparative encyclopedia of DNA elements in the mouse genome. Nature.

[21] Fagerberg L., Hallstrom B.M., Oksvold P., Kampf C., Djureinovic D., Odeberg J. (2014). Analysis of the human tissue-specific expression by genome-wide integration of transcriptomics and antibody-based proteomics. Mol. Cell Proteomics.

[22] Cho C.H., Patel S., Rajbhandari P. (2023). Adipose tissue lipid metabolism: lipolysis. Curr. Opin. Genet. Dev..

[23] Choi E.J., Jeon C.H., Lee I.K. (2022). Ferric ammonium citrate upregulates PD-L1 expression through generation of reactive oxygen species. J. Immunol. Res..

[24] Joffin N., Gliniak C.M., Funcke J.B., Paschoal V.A., Crewe C., Chen S. (2022). Adipose tissue macrophages exert systemic metabolic control by manipulating local iron concentrations. Nat. Metab..

[25] Higashida K., Takeuchi N., Inoue S., Hashimoto T., Nakai N. (2020). Iron deficiency attenuates catecholamine-stimulated lipolysis *via* downregulation of lipolysis-related proteins and glucose utilization in 3T3-L1 adipocytes. Mol. Med. Rep..

[26] Yook J.S., You M., Kim J., Toney A.M., Fan R., Puniya B.L. (2021). Essential role of systemic iron mobilization and redistribution for adaptive thermogenesis through HIF2-alpha/hepcidin axis. Proc. Natl. Acad. Sci. U. S. A..

[27] Fischer C., Volani C., Komlodi T., Seifert M., Demetz E., Valente de Souza L. (2021). Dietary iron overload and Hfe(-/-) related hemochromatosis alter hepatic mitochondrial function. Antioxidants (Basel).

[28] Deschemin J.C., Ransy C., Bouillaud F., Chung S., Galy B., Peyssonnaux C. (2023). Hepcidin deficiency in mice impairs white adipose tissue browning possibly due to a defect in *de novo* adipogenesis. Sci. Rep..

[29] Blankenhaus B., Braza F., Martins R., Bastos-Amador P., Gonzalez-Garcia I., Carlos A.R. (2019). Ferritin regulates organismal energy balance and thermogenesis. Mol. Metab..

[30] Silva G.D.N., Amato A.A. (2022). Thermogenic adipose tissue aging: mechanisms and implications. Front. Cell Dev. Biol..

[31] Fu W., Liu Y., Sun C., Yin H. (2019). Transient p53 inhibition sensitizes aged white adipose tissue for beige adipocyte recruitment by blocking mitophagy. FASEB J..

[32] Wu Q., Wang H., An P., Tao Y., Deng J., Zhang Z. (2015). HJV and HFE play distinct roles in regulating hepcidin. Antioxid. Redox Signal..

[33] Lettieri Barbato D., Aquilano K., Baldelli S., Cannata S.M., Bernardini S., Rotilio G. (2014). Proline oxidase-adipose triglyceride lipase pathway restrains adipose cell death and tissue inflammation. Cell Death Differ..

[34] Khan S.A., Sathyanarayan A., Mashek M.T., Ong K.T., Wollaston-Hayden E.E., Mashek D.G. (2015). ATGL-catalyzed lipolysis regulates SIRT1 to control PGC-1alpha/PPAR-alpha signaling. Diabetes.

[35] Rumberger J.M., Peters T., Burrington C., Green A. (2004). Transferrin and iron contribute to the lipolytic effect of serum in isolated adipocytes. Diabetes.

[36] Yang Loureiro Z., Solivan-Rivera J., Corvera S. (2022). Adipocyte heterogeneity underlying adipose tissue functions. Endocrinology.

[37] Mahmoud M., Abdel-Rasheed M. (2023). Influence of type 2 diabetes and obesity on adipose mesenchymal stem/stromal cell immunoregulation. Cell Tissue Res..

[38] Wang T., Sharma A.K., Wolfrum C. (2022). Novel insights into adipose tissue heterogeneity. Rev. Endocr. Metab. Disord..

[39] Shinde A.B., Nunn E.R., Wilson G.A., Chvasta M.T., Pinette J.A., Myers J.W. (2023). Inhibition of nucleotide biosynthesis disrupts lipid accumulation and adipogenesis. J. Biol. Chem..

[40] Yang X., Liu Q., Li Y., Tang Q., Wu T., Chen L. (2020). The diabetes medication canagliflozin promotes mitochondrial remodelling of adipocyte *via* the AMPK-Sirt1-Pgc-1alpha signalling pathway. Adipocyte.

[41] Tan T.C., Crawford D.H., Jaskowski L.A., Murphy T.M., Heritage M.L., Subramaniam V.N. (2011). Altered lipid metabolism in Hfe-knockout mice promotes severe NAFLD and early fibrosis. Am. J. Physiol. Gastrointest. Liver Physiol..

[42] Sun Z., Pan X., Tian A., Surakka I., Wang T., Jiao X. (2023). Genetic variants in HFE are associated with non-alcoholic fatty liver disease in lean individuals. JHEP Rep..

[43] Barton J.C., Barton J.C., Adams P.C., Acton R.T. (2016). Undiagnosed diabetes and impaired fasting glucose in HFE C282Y homozygotes and HFE wild-type controls in the HEIRS Study. BMJ Open Diabetes Res. Care.

[44] Gabrielsen J.S., Gao Y., Simcox J.A., Huang J., Thorup D., Jones D. (2012). Adipocyte iron regulates adiponectin and insulin sensitivity. J. Clin. Invest..

[45] Kim M., Kim Y.H., Min S., Lee S.M. (2022). High iron exposure from the fetal stage to adulthood in mice alters lipid metabolism. Nutrients.

[46] Dongiovanni P., Ruscica M., Rametta R., Recalcati S., Steffani L., Gatti S. (2013). Dietary iron overload induces visceral adipose tissue insulin resistance. Am. J. Pathol..

[47] Suga H., Matsumoto D., Eto H., Inoue K., Aoi N., Kato H. (2009). Functional implications of CD34 expression in human adipose-derived stem/progenitor cells. Stem Cells Dev..

[48] Raajendiran A., Ooi G., Bayliss J., O'Brien P.E., Schittenhelm R.B., Clark A.K. (2019). Identification of metabolically distinct adipocyte progenitor cells in human adipose tissues. Cell Rep..

[49] Shi L., Tao Z., Zheng L., Yang J., Hu X., Scott K. (2023). FoxO1 regulates adipose transdifferentiation and iron influx by mediating Tgfbeta1 signaling pathway. Redox Biol..

[50] Suzuki T., Komatsu T., Shibata H., Tanioka A., Vargas D., Kawabata-Iwakawa R. (2023). Crucial role of iron in epigenetic rewriting during adipocyte differentiation mediated by JMJD1A and TET2 activity. Nucleic Acids Res..

[51] Liu J., Wu X., Wang H., Wei J., Wu Q., Wang X. (2021). HFE inhibits type I IFNs signaling by targeting the SQSTM1-mediated MAVS autophagic degradation. Autophagy.

[52] Ding H., Chen S., Pan X., Dai X., Pan G., Li Z. (2021). Transferrin receptor 1 ablation in satellite cells impedes skeletal muscle regeneration through activation of ferroptosis. J. Cachexia Sarcopenia Muscle.

[53] Aune U.L., Ruiz L., Kajimura S. (2013). Isolation and differentiation of stromal vascular cells to beige/brite cells. J. Vis. Exp..

[54] Sanchez-Ceinos J., Guzman-Ruiz R., Rangel-Zuniga O.A., Lopez-Alcala J., Moreno-Cano E., Del Rio-Moreno M. (2021). Impaired mRNA splicing and proteostasis in preadipocytes in obesity-related metabolic disease. Elife.

[55] Moos T., Mollgard K. (1993). A sensitive post-DAB enhancement technique for demonstration of iron in the central nervous system. Histochemistry.

