# Supplementary material for: Iron supplementation and iron accumulation promote adipocyte thermogenesis through PGC1α-ATGL–mediated lipolysis
Source: J Biol Chem. 2024 Aug 17;300(9):107690. doi: 10.1016/j.jbc.2024.107690 (PMC11420453; doi:10.1016/j.jbc.2024.107690)
Supplement: Supporting information figure legends [file mmc1.docx]

**Supporting information**

**Supporting information Figure 1 for Figure 3. Iron metabolism involved in cold induced adipose thermogenesis process.** (A) Biological process (BP) and (B) cellular component (CC) analysis of GO analysis in iWAT from 10~15-week-old WT mice between room temperature and cold stimulation (n=3/group); (C) Biological process (BP) and (D) molecular function (MF) analysis of GO analysis in BAT from 10~15-week-old WT mice at room temperature and cold exposure; (E) Volcano map of differential genes of BAT from 10~15-week-old WT mice at room temperature and cold exposure (n=3/group); (F) Heat map of iron metabolism related genes expression in BAT and iWAT (G) from 10~15-week-old WT mice at room temperature and cold stimulation (n=3/group).

**Supporting information Figure 2 for Figure 4. Iron accumulation caused by *Hfe* deficiency regulate adipose thermogenesis.** (A) Nucleic acid gel electrophoresis for genotyping in WT and *Hfe*^-/-^ group; (B) Serum iron level and (C) tissue iron level from 30~35-week-aged WT and *Hfe*^-/-^ at RT and cold exposure (n=3/group); (D) Protein expression analysis of Ucp1, Tfr1, Fth, PGC-1α and Tubulin of BAT for figure 4E; (E) Protein expression analysis of Ucp1, Tfr1, Fth, PGC1α and Tubulin of iWAT for figure 4F; (F) RNA-seq heatmap of iWAT from WT and *Hfe*^-/-^ mice at RT (n=3/group); (G) Volcano plot of different genes of iWAT in WT and *Hfe*^-/-^ group at RT; (H) KEGG analysis of different genes of iWAT in WT and *Hfe*^-/-^ group at RT; (I) GSEA analysis of oxidative phosphorylation, (J) Fatty acid β-oxidation and (K) Mitochondrial gene expression of iWAT between WT and *Hfe*^-/-^ at RT; (L) Protein immunoblot (Tubulin, Mitochondrial-complex) of iWAT from WT and *Hfe*^-/-^ mice at RT (n=3/group); (M) Heatmap of tgfβ1 signaling pathway relative genes of iWAT between WT and *Hfe*^-/-^ at RT. Data show as the mean ± SD, **P* < 0.05, ** *P* < 0.01 and *** *P* < 0.001, N.S., not significant. Unpaired student’s t test for two group comparison.

**Supporting information Figure 3 for Figure 4. Iron accumulation caused by *Hfe* deficiency regulate adipose thermogenesis.** (A) RNA-seq heatmap of BAT from 10~15-week-old WT and *Hfe*^-/-^ group in RT (n=3/group); (B) Volcano plot of different genes of BAT from 10~15-week-old WT and *Hfe*^-/-^ group in RT.

**Supporting information Figure 4 for Figure 5. Impact of *Hfe* Deficiency and Iron Supplementation on SVFs and Adipocytes *in vitro*.** (A) Protein analysis of immunoblots for figure 5B; (B) Protein immunoblots of p-AMPK, Ampk, p-HSL, HSL of iWAT primary adipocyte from WT and *Hfe*^-/-^ after adipogenic differentiation (n=3/group); (C) qPCR analysis of gene expression in BAT primary adipocyte from WT and *Hfe*^-/-^ after adipogenic differentiation (n=3/group) and (D) Protein immunoblots of BAT primary adipocyte from WT and *Hfe*^-/-^ after adipogenic differentiation (n=3/group); (E) Representative images of fluorescence staining of BAT primary adipocyte from WT and *Hfe*^-/-^ after 7days differentiation; (F) *Hfe*, *Atgl* relative mRNA expression of iWAT between 30~35-week-aged WT and *Hfe*^-/-^ at RT (n=6 mice/group); (G) Representative immunoblots (PGC-1α, ATGL, Tubulin) in iWAT from 30~35-week-aged WT and *Hfe*^-/-^ mice at RT ; (H) Protein analysis of ATGL in iWAT from 30~35-week-aged WT and *Hfe*^-/-^ mice at RT (n=3 mice/group). Data show as the mean ± SD, unpaired student’s t test for two group comparison. **P* < 0.05, ** *P* < 0.01 and *** *P* < 0.001, N.S., not significant.
