# Supplementary material for: Iron supplementation and iron accumulation promote adipocyte thermogenesis through PGC1α-ATGL–mediated lipolysis
Source: J Biol Chem. 2024 Aug 17;300(9):107690. doi: 10.1016/j.jbc.2024.107690 (PMC11420453; doi:10.1016/j.jbc.2024.107690)
Supplement: Supporting information table [file mmc2.docx]

**Supporting information table 1. qPCR Primer list**

| Gene | Forward Primer | Reverse Primer |
| --- | --- | --- |
| *Rps18* | CGCCATGTCTCTAGTGATCC | GGTCGATGTCTGCTTTCCTC |
| *Ucp1* | ATACTGGCAGATGACGTCCC | CGAGTCGCAGAAAAGAAGCC |
| *Dio2* | CAGTGTGGTGCACGTCTC | TGAACCAAAGTTGACCACCAG |
| *Prdm16* | ACATTCCAATCCCACCAGAC | CCAAGTCTTCAGAGATCTGCTTT |
| *Cox7a1* | AGCTGCTGAGGACGCA | GCTTCTGCTTCTCTGCCAC |
| *Cox8b* | TTCCCAAAGCCCATGTCTCT | GGCTAAGACCCATCCTGCT |
| *Cidea* | ATACATCCAGCTCGCCCTTT | ACTTACTACCCGGTGTCCAT |
| *Ppargc1α* | CTCTGGAACTGCAGGCCTAA | TGCCTTGGGTACCAGAACA |
| *Hfe* | TCTTCCCCCAGAACATCACT | CTCATCCCCGTTAGGTAGCA |
| *Tfrc* | TCGTACAGCAGCGGAAGT | TCTCCACGAGCGGAATACAG |
| *Slc11a2* | CCAGGATGTGGAGCACCTA | GCTTGTGAACGTGAGGATGG |
| *Slc40a1* | TTGTGGCAGGAGAAAACAGG | GCCAATGACTGGAGAACCAA |
| *Fth1* | GTGCGCCAGAACTACCAC | AGCCACATCATCTCGGTCA |
| *Ftl1* | CTACCTCTCTCTGGGCTTCTT | ATGGCCAAGGCAGCTTC |
| *Gatm* | ATCCTCTGCCCAAGGACTG | ACGGTCACTCCTTCCATTGATA |
| *Atgl*  (*Pnpla2*) | ACTGTGGCCTCATTCCTCC | GGGACACTGTGATGGTATTCTTCA |
| *Ebf2* | CGATGCTGTGAGAAGAAGAGC | CTTCAGATGGGTCAAGCCTTC |
| *Slc2a4* | GTTGGTCTCGGTGCTCTTAGT | TGAAGAGCTCTGCCACAATGA |
| *Acc1*  (*Acaca*) | AAGCCCAGCTCTGGAACA | AGTCTGTCCAGCCAGCC |
| *Fasn* | AACCTGGCCATGGTTTTGAG | GCCTGCGCTGTTCACATATA |
| *Pparγ* | TCTCCAGCATTTCTGCTCCA | GGCCATGAGGGAGTTAGAAGG |
| *Dclk1* | TGAGCATCCCTGGGTTAATGAT | GAAACTCCTGCTGCAGTGC |
| *En1* | ACAGCAACCCCTAGTGTGG | TAGCGGTTTGCCTGGAACT |
| *Kit* | AGAGATTTGGCAGCCAGGA | TCTCTGGTGCCATCCACTTC |
| *Thy1* | CTGCTCTCAGTCTTGCAGGT | GTGTGCTCGGGTATCCCAA |
| *Ly6a* | TCAATTACCTGCCCCTACCC | CAGAGGTCTTCCTGGCAACA |
| *Cd34* | CCAGGGTATCTGCCTGGAAC | TCAGCCTCCTCCTTTTCACA |
| *Cd36* | TTCAATGGAAAGGATAACATAAGCAAAG | CTGTGCCATTAATCATGTCGCA |

**Supplementary table 2. Genotyping primer list**

| Gene | Forward Primer | Reverse Primer |
| --- | --- | --- |
| *Hfe_WT* | ATCAAATGGCATCTCTGGCA | GTGGCGAGTCACTTTCACCA |
| *Hfe_KO* | AGTTGGGAGTGGTGTCCGA | CTAGCTTCGGCCGTGACG |
